# Inhibitor of Hyaluronic Acid Synthesis 4-Methylumbelliferone Suppresses the Secretory Processes That Ensure the Invasion of Neutrophils into Tissues and Induce Inflammation

**DOI:** 10.3390/biomedicines10020314

**Published:** 2022-01-28

**Authors:** Svetlana I. Galkina, Natalia V. Fedorova, Alexander L. Ksenofontov, Ekaterina A. Golenkina, Marina V. Serebryakova, Vladimir I. Stadnichuk, Ludmila A. Baratova, Galina F. Sud’ina

**Affiliations:** 1 A.N. Belozersky Institute of Physico-Chemical Biology, M.V. Lomonosov Moscow State University, 119991 Moscow, Russia; fedorova@genebee.msu.ru (N.V.F.); ksenofon@belozersky.msu.ru (A.L.K.); golyesha@mail.ru (E.A.G.); mserebr@mail.ru (M.V.S.); baratova@genebee.msu.ru (L.A.B.); 2 Physical Department, M.V. Lomonosov Moscow State University, 119991 Moscow, Russia; stadn@polly.phys.msu.ru

**Keywords:** 4-methylumbelliferone, neutrophil, adhesion, secretion, hydroxylysine, cytoneme, exocytosis, LPS, nitric oxide, actin depolymerization

## Abstract

Integrin-dependent adhesion of neutrophils to tissue, accompanied by the development of neutrophil-induced inflammation, occurs both in the focus of infection and in the absence of infection in metabolic disorders such as reperfusion after ischemia, diabetes mellitus, or the development of pneumonia in patients with cystic fibrosis or viral diseases. Hyaluronic acid (HA) plays an important role in the recruitment of neutrophils to tissues. 4-methylumbilliferon (4-MU), an inhibitor of HA synthesis, is used to treat inflammation, but its mechanism of action is unknown. We studied the effect of 4-MU on neutrophil adhesion and concomitant secretion using adhesion to fibronectin as a model for integrin-dependent adhesion. 4-MU reduced the spreading of neutrophils on the substrate and the concomitant secretion of granule proteins, including pro-inflammatory components. 4-MU also selectively blocked adhesion-induced release of the free amino acid hydroxylysine, a product of lysyl hydroxylase, which can influence cell invasion by modifying the extracellular matrix. Finally, 4-MU inhibited the formation of cytonemes, the extracellular membrane secretory structures containing the pro-inflammatory bactericides of the primary granules. The anti-inflammatory effect of 4-MU may be associated with the suppression of secretory processes that ensure the neutrophil invasion and initiate inflammation. We suggest that HA, due to the peculiarities of its synthesis, can promote the release of secretory carriers from the cell and 4-MU can block this process.

## 1. Introduction

The study of the mechanisms of adhesion and secretion of neutrophils is necessary to solve such problems as the prevention and treatment of inflammatory processes caused by the invasion of neutrophils in the tissue and concomitant secretion. Neutrophils have the ability to migrate out of the bloodstream and penetrate into tissues at the site of infection, where they adhere and capture and kill microorganisms by releasing bactericidal components of intracellular granules and reactive oxygen species [1,2]. Aggressive bactericidal secretion of neutrophils also penetrates the surrounding tissues, where it initiates inflammatory processes. Integrin-dependent tissue adhesion of neutrophils and neutrophil-induced inflammation also occur in the absence of infection in certain metabolic diseases, such as reperfusion after ischemia [3] and diabetes [4]. The lungs are one of the main reservoirs of neutrophils, where neutrophils, along with other lung cells, play an important role in the development of pneumonia [5,6]. The participation of neutrophils in the development of pneumonia in cystic fibrosis, and chronic obstructive pulmonary, as well as other pulmonary, pathologies has been shown [5,7,8,9] and, apparently, in acute respiratory distress syndrome in coronavirus disease [10].

Hyaluronic acid (HA) and the interaction of HA with its main ligand, CD44, plays an important role in the recruitment of neutrophils into tissues [11,12,13]. Neither neutrophil flux nor rolling velocities decreased in CD44^−/−^ mice, but the number of attached leukocytes in the venule decreased by 65% compared to wild-type mice [11]. Hyaluronidase, the enzyme that destroys HA, decreases neutrophil recruitment and adhesion to tissues [11,12,13,14]. An inhibitor of HA synthesis, 4-methylumbelliferone (4-MU), has been shown to block HA production in many cell lines and tissue types both in vivo and in vitro. 4-MU depletes the cellular pool of UDP-glucuronic acid, a precursor of HA synthesis, and decreases mRNA levels of HA synthase 2 and 3 [15,16]. 4-MU has recently been used as a therapeutic agent in the treatment of cancer and inflammation. It has been shown that 4-MU inhibits migration and adhesion to tissues of various types of cancer and other cells [16,17,18,19]. 4-MU prevents inflammatory lung injury in mouse models of staphylococcal enterotoxin-mediated [20] and lipopolysaccharide-mediated acute lung injury [21], and shows protective effect against renal ischemia-reperfusion injury and LPS-induced acute kidney injury [22,23]. Whether the anti-inflammatory effect of 4-MU is related to neutrophil recruitment and neutrophil-induced inflammation remains to be seen.

In this work, we studied the effect of 4-MU on the secretory processes of neutrophils during adhesion to fibronectin. In previous studies, we have shown that neutrophil adhesion to fibronectin changes the profile of free amino acid secretion by neutrophils, namely, it sharply and selectively stimulates the release of hydroxylysine into the extracellular medium [24,25]. Hydroxylysine is formed by the action of the enzyme lysyl hydroxylase (LH 1-3 or procollagen lysine, 2-oxoglutarate-5-dioxygenase, PLOD 1-3). LH modifies collagen lysine residues intracellularly and extracellularly, thereby reorganizing the extracellular matrix [26]. Extracellular localization and activity of LH is characteristic of tumor cells [27,28,29]. Increased expression and secretion of LH into the extracellular medium has been demonstrated for cells of lung cancer, glioma, glioblastoma and pancreatic duct adenocarcinoma [30,31,32,33,34]. Increased expression of these enzymes clearly correlates with the formation of metastases and poor prognosis in cancer patients. Based on the similarity of the processes of recruiting neutrophils in the tissues of the body and the formation of metastases by tumor cells, we assumed that the release of hydroxylysine and activation of LH plays an important role in the processes of invasion and adhesion of neutrophils in the tissue.

Adhesion of neutrophils to fibronectin is also accompanied by the secretion of proteins, including lactoferrin, myeloperoxidase, albumin, lipocalin associated with neutrophil gelatinase, proteins S100A8 and S100A9, and lysozyme [35]. The hormones insulin and 17 ß-estradiol, as well as the classic secretory stimuli fMLP, PMA, or LPS, in addition to proteins secreted by control cells, initiated the secretion of metalloproteinases (MMP-9 and MMP-8), which support the migration and adhesion of neutrophils by modulating the extracellular matrix and basement membranes. LPS and the hormone glucagon stimulated the secretion of primary granule pro-inflammatory bactericides such as cathepsin G and defensins [36,37]. Depolymerization of the actin cytoskeleton with cytochalasin D disrupted MMP secretion, but initiated the release of cathepsin G and other granular bactericides [37,38].

In blood vessels, blood pressure and a network of mediators, among which nitric oxide (NO) occupies a special place, prevent integrin-dependent leukocyte adhesion. We have shown that the protective effect of NO in vascular pathologies may be associated with a fundamental change in the mechanism of adhesion and secretion of neutrophils. [39,40]. NO initiated the formation of cytonemes on the surface of neutrophils—filamentous structures consisting of membrane vesicles and tubules of the same diameter, containing secretory bactericides of neutrophils. These secretory structures establish adhesive interactions of neutrophils with substrates, other cells, and bacteria at a distance [35,41]. Along with NO, actin-depolymerizing alkaloids of invading microbes, such as the mold alkaloid cytochalasin D or staurosporine, the alkaloid Streptomyces staurosporeus, appear to be natural initiators of cytoneme formation in neutrophils [41,42,43].

In patients with metabolic disorders (surgical patients with reperfusion after ischemia, in patients with diabetes mellitus, or in patients with pneumonia provoked by cystic fibrosis or viral diseases), neutrophil infiltration into tissues, leading to severe inflammatory processes, occurs and in the absence of infection, as a result of which the use of antibiotics becomes ineffective. Therefore, the study of the possibilities of prevention and therapy of inflammatory processes with drugs that regulate invasion and secretion of neutrophils is becoming increasingly relevant and important. In this article, we investigated whether 4-MU (7-hydroxy-4-methylcoumarin), also known as hymecromone, can influence neutrophil-induced inflammation. We studied the effect of 4-MU on neutrophil adhesion and concomitant secretion of free amino acids and proteins, as well as on NO- and staurosporine-induced cytoneme formation in human neutrophils during adhesion to fibronectin. The effect of 4-MU on the morphology of attached neutrophils and cytoneme formation was studied using scanning electron microscopy. The secreted proteins were separated by polyacrylamide gel electrophoresis and identified by mass spectrometry. Free amino acids secreted during adhesion were identified by amino acid analysis.

## 2. Materials and Methods

### 2.1. Materials

Hank’s balanced salt solution (HBSS) without sodium bicarbonate, Dulbecco’s Phosphate-Buffered Saline (D-PBS) without CaCl_2_, 4-methylunbelliferone (4-MU), lipopolysaccharides from Salmonella enterica serotype typhimurium (LPS), diethylamine NONOate sodium salt hydrate, thiol protease inhibitor E64, and human plasma fibronectin were obtained from Merck KGaA (Darmstadt, Germany). Ficoll-Paque was obtained from Pharmacia (Uppsala, Sweden). Coomassie Brilliant Blue G-250 was obtained from Serva (Heidelberg, Germany); PMSF from MP Biomedical (Irvine, CA, USA); trypan blue from Honeywell Fluka (Charlotte, NC, USA); and glutaraldehyde from Ted Pella (Redding, CA, USA). Trypsin was from Promega (Madison, WI, USA), and carboxy-H_2_DCF-DA from Molecular Probe (Eugene, OR, USA). Analytical chromatography solvents: eluent MCI Buffer L-8800-PH-1–4 and ninhydrin coloring solution kit for Hitachi 29970501 from FUJIFILM Wako Chemicals GmbH (Richmond, VA, USA).

### 2.2. Neutrophil Preparation

Neutrophils were isolated from the citrate-anticoagulated blood of healthy volunteers after obtaining informed consent. None of the donors had been on medication for at least two weeks prior to blood donation. The experiments were approved by the Bioethics Commission of M.V. Lomonosov Moscow State University, application # 6-h version 3, approved during the Bioethics Commission meeting # 131-days held on 31 May 2021. The procedure for cell separation is described in detail earlier [25]. Briefly, after primary erythrocytes sedimentation with 3% T-500 Dextran, neutrophils were isolated from leukocyte-rich plasma by centrifugation through Ficoll-Paque at a density of 1.077 g/mL, followed by hypotonic lysis of the remaining erythrocytes and double washing in PBS. Neutrophils were then suspended at 10^7^ cells/mL (96–97% purity, 98–99% viability) in D-PBS containing 1 mg/mL glucose and stored at room temperature.

### 2.3. Adhesion of Neutrophils to the Fibronectin-Coated Substrate

Greiner CELLSTAR^®^ 6-well culture plates (Greiner Bio-One GmbH, Kremsmünster, Austria) were incubated with fibronectin (5 µg/mL) for 2 h and thoroughly washed. Attachment of neutrophils (3 × 10^6^ cells/mL HBSS supplemented with 10 mM HEPES [pH 7.35]) occurred within 25 min at 37 °C in a 5% CO_2_ incubator. LPS, diethylamine NONOate and 4-MU were added to the cells before incubation. At the end of the exposure, samples of the extracellular medium were taken and mixed with: 5 mM EDTA, inhibitor of metalloproteinase; 200 µm PMSF, inhibitor of serine proteinases; 10 µM E64, inhibitor of cysteine proteinases; and 0.025% sodium azide, myeloperoxidase inhibitor. Unattached neutrophils were removed by centrifugation (5 min at 400× *g*). For amino acid analysis extracellular medium samples from three identical wells were pooled. Samples from six identical wells were combined to determine the protein content of neutrophils secretion. To exclude the possibility that the appearance of proteins or amino acids in the extracellular medium was the result of cell destruction, neutrophils were stained with trypan blue after collecting the extracellular medium. Trypan blue stains dead cells, but does not penetrate viable cells. The percentage of dead cells from the total number of cells was counted (3000 cells were counted for each group).

### 2.4. Study of Neutrophil Morphology with Scanning Electron Microscopy

Cell morphology and formation of cytonemes were studied on cells attached to fibronectin-coated coverslips. The procedure for covering with fibronectin was identical to that for culture plates. The prepared coverslips were placed in the wells of the culture plate, and neutrophils (1.5 × 10^6^ cells/mL HBSS supplemented with 10 mM HEPES [pH 7.35]) were incubated for 25 min at 37 °C in a 5% CO_2_ atmosphere. LPS (10 μg/mL), 1 mM diethylamine NONOate, and 0.5 mM 4-MU were added to the cells at the initial step. Attached cells were fixed in 2.5% glutaraldehyde in HBSS/HEPES supplemented with 5 mM EDTA and 0.5 mM PMSF. Additionally, 1% osmium tetroxide in 0.1 M sodium cacodylate containing 0.1 M sucrose at pH 7.3 was used as a secondary fixative. Samples were then dehydrated in a series of acetones (10–100%) and dried in a Balzer apparatus at the critical point with liquid CO_2_ as a transition liquid. Sputter coating of gold/palladium was used followed by samples examination at 15 kV with a scanning electron microscope Camscan S-2. The area occupied by the cells on the substrate was measured quantitatively using an ImageJ-win64 software in scanning electron microscopy images.

### 2.5. Quantification of Neutrophil Attachment

Neutrophils (2 × 10^5^ cells/probe) were attached to fibronectin-coated 96-well plates for 30 min at 37 °C in 5% CO_2_ under control conditions or in the presence of 4-MU (0.5–1 mM). After supernatants were removed, weakly attached cells were removed by washing with warm PBS. The percent of firmly attached cells was determined by the method of Ngo et al. [44,45], based on the measurement of absorption of 2, 3-diaminophenazine, colored product of MPO-catalyzed oxidation of o-phenylenediamine dihydrochloride (OPD) by H_2_O_2_. H_2_O_2_ (4 mM final concentration) with 5.5 mM OPD in permeabilizing buffer (67 mM Na_2_HPO_4_, 35 mM citric acid, and 0.1% Triton X-100) was added to attached neutrophils for 5 min. The reaction was stopped by adding of 1M H_2_SO_4_. The absorption was measured at a wavelength of 490 nm and compared with the calibration values.

### 2.6. Separation of Proteins by Electrophoresis in Polyacrylamide Gel

Proteins were extracted from samples of the extracellular medium with chloroform–methanol mixture. The chloroform phase was collected and subjected to electrophoresis after evaporation of the solvent as was published [35]. Proteins were separated by one-dimensional electrophoresis in the presence of sodium dodecyl sulfate on a 15% polyacrylamide gel under non-reducing conditions in a Mini-PROTEAN 3 cell (Bio-Rad). Protein bands were stained with Coomassie brilliant blue G-250.

### 2.7. Mass Spectrometry Identification of Proteins

From each protein band, gel pieces were cut out, which were washed, dehydrated, air-dried, and subjected to trypsin digestion directly in the gel. The resulting peptides were extracted with 0.5% trifluoroacetic acid. Aliquots were taken from each sample, mixed on a steel target with 2,5-dihydroxybenzoic acid, dried, and subjected to mass spectrometric analysis as was published [35]. Matrix assisted laser desorption ionization mass spectrometry (MALDI-MS) analysis of proteins was performed with a MALDI-ToF-ToF mass spectrometer Ultraflextreme (Bruker, Karlsruhe, Germany). Identification of proteins was carried out by a peptide fingerprint search using Mascot software 2.5.01 (http://www.matrixscience.com, accessed on 3 January 2021), SwissProt database through the mammalian proteins.

### 2.8. Amino Acid Analysis and Sample Preparation

After concentrating the samples of the extracellular medium, the proteins were precipitated with sulfosalicylic acid and removed by centrifugation. Supernatants were purified by centrifugation through Vivaspin 500 Membrane 3000 PES MWCO ultrafilters (Sartorius, Goettingen, Germany) and subjected to amino acid analysis on an L-8800 amino acid analyzer (Hitachi, Tokyo, Japan) in the standard mode. Amino acids were quantified in 100 μL of the extracts injected to an ion-exchange column 2622SC(PH) (Hitachi, Ltd., P/N 855-3508, 4.6 × 80 mm), eluted by the step gradient of sodium-acetate buffers according to the previously published method [24].

### 2.9. Statistics

The determination of the amino acid and protein composition of neutrophil secretion, as well as the study of the morphology of attached neutrophils, was repeated three times using the blood of different donors. Results are presented as mean ± standard error of the mean. Statistical significance was assessed using GraphPadPrism7 software. An unpaired *t*-test was applied to estimate cell area. To quantify the amino acid profile of secretion, a two-way ANOVA with Tukey’s multiple comparison test was used.

## 3. Results

### 3.1. Effect of 4-Methylumbelliferone on Neutrophil Adhesion and Spreading over Fibronectin-Coated Substrata

**Figure 1:**
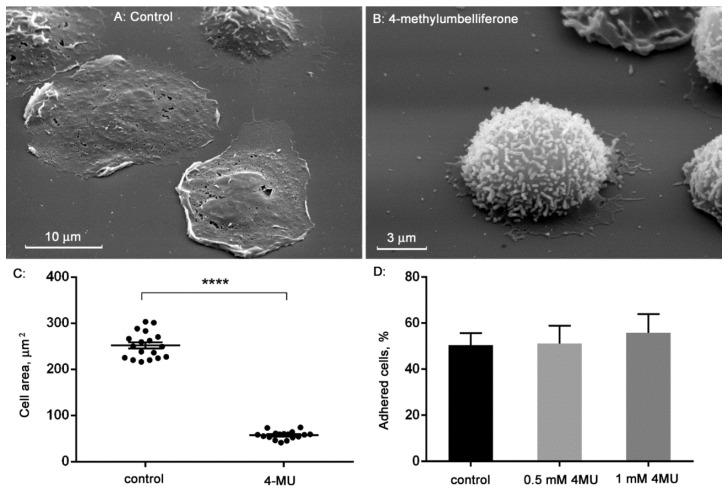
4-methylumbelliferone inhibits the spreading of neutrophils on substrates coated with fibronectin. Images of human neutrophils that were adhered to fibronectin-coated substrates for 25 min under control conditions (**A**) or in the presence of 0.5 mM 4-MU (**B**) were obtained by scanning electron microscopy. Images are typical images observed in three independent experiments. Area occupied by neutrophils adhered for 25 min to a fibronectin-coated substrate under control conditions or in the presence of 0.5–1 mM 4-MU. **** *p* < 0.0001, as shown by the unpaired *t*-test (*n* = 18) (**C**). The number of firmly attached neutrophils as a percentage of the total number of cells after attachment of neutrophils to fibronectin for 25 min under control conditions or in the presence of 4-MU (**D**).

We compared the morphology of neutrophils that were attached to fibronectin-coated substrate under control conditions or in the presence of 4-MU by scanning microscopy. Control neutrophils adhered and spread well over fibronectin. Spread neutrophils have a smooth surface (Figure 1A). In the presence of 4-MU, the spreading of neutrophils was markedly suppressed. The cells partially retained the round shape characteristic of neutrophils in suspension. The surface of neutrophils in the presence of 4-MU was covered with numerous short microprotrusions (Figure 1B). 4-MU significantly reduced the area occupied by neutrophils on the substrate, as measured in SEM images of control and 4-MU treated cells using ImageJ-win64 software (Figure 1C). The average area of control cells was 252 μm^2^, which is statistically significant, more than four times the 57 μm^2^, the average area of cells attached in the presence of 4-MU (Figure 1C).

Our data indicated that 0.5–1 mM 4-MU did not affect cell attachment to fibronectin-coated substrata (Figure 1D). The number of firmly attached control and 4-MU-treated neutrophils were compared after double washing with warm PBS to remove free-floating or weakly attached cells. Analogous experiments also did not reveal the statistically significant effect of 4-MU on neutrophil attachment to fibrinogen (data not shown). 

### 3.2. Effect of 4-Methylumbelliferone on Protein Secretion by Neutrophils during Adhesion to Fibronectin under Control Conditions and in the Presence of LPS

We studied the effect of 4-MU on the composition of protein secretion by neutrophils during adhesion to fibronectin. Extracellular samples were taken from neutrophils after 25 min adhesion to fibronectin coated substrates. Proteins were extracted with a chloroform–methanol mixture and separated by electrophoresis. Silver staining of electrophoretic gels showed that neutrophils secreted numerous proteins during adhesion (data not shown), but silver staining did not allow protein identification by mass spectrometry. The electrophoretic gels were stained with Coomassie brilliant blue, which stained only basic, but not all secreted proteins. Coomassie brilliant blue staining allowed us to identify secreted proteins and to establish protein profiles of neutrophil secretion specific to each treatment [35,37]. To demonstrate that proteins were released by viable cells, we stained neutrophils after collection of the extracellular medium with trypan blue. The percent of stained (dead) cells did not exceed 1–2% in all preparations.

**Figure 2:**
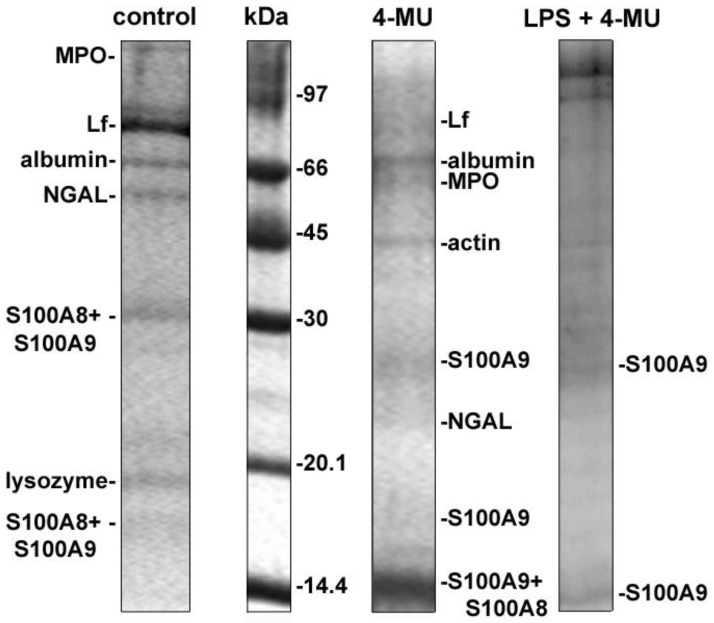
4-MU inhibits the secretion of proteins by neutrophils during adhesion to fibronectin. Human neutrophils were attached to fibronectin coated substrates during 25 min incubation under control conditions or in the presence of 1 mM 4-MU. The extracellular medium was collected, proteins were extracted, subjected to separation in 15% SDS-PAGE, and stained with Coomassie brilliant blue. Images are typical protein profiles observed in three independent experiments.

The secretion profile includes proteins that were reliably identified in three similar experiments. The secretory protein profile of neutrophils, which were attached to fibronectin under control conditions, included components of primary (MPO) and secondary (LF, NGAL, lysozyme) secretory granules of neutrophils, albumin, which is localized in secretory vesicles and cytosolic proteins, S100A8 and S100A9 (Figure 2). This protein profile is consistent with the previously published protein profile of control neutrophils [25,35], indicating that the composition of protein secretion during cell adhesion is a stable specific property of neutrophils. As shown previously and recently, lactoferrin (Lf), a component of secondary intracellular granules, has been identified in the dominant protein band (Figure 2).

When neutrophils were attached to fibronectin in the presence of 4-MU, the granular proteins Lf and NGAL were present in trace amounts, and another granular protein lysozyme was not identified. The protein profile of secretion included proteins S100A9 and S100A8, MPO, and, in addition to control cells, included the cytosolic protein actin (Figure 2, Table 1). All proteins in the list were identified with statistically significant score. These data show that 4-MU reduces the release of neutrophilic granule components, but initiates the release of cytosolic proteins during adhesion. Bacterial lipopolysaccharides (LPS or endotoxins) of the outer membrane of Gram-negative bacteria are the main inducer of the host’s response to infection and an effective stimulator of innate immunity, typical representatives of which are neutrophils [46]. The source of LPS can be bacteria that have entered the body from the environment, or bacteria in the gastrointestinal tract. As previously published, the secretory protein profile of neutrophils that were attached to fibronectin in the presence of LPS contained the same proteins as control cells, and in addition: (i) MMP-9, a component of tertiary granules; (ii) cathepsin G and defensins, potent primary granule bactericides; and (iii) a number of cytosolic proteins such as actin, the glycolytic enzyme glucose-6-phosphate dehydrogenase, and S100 proteins [37,47]. When neutrophils were attached to fibronectin in the presence of LPS and 4-MU, neutrophil secretion was largely inhibited (Figure 2). The secreted proteins were present in the extracellular environment in trace amounts, and only one protein, the cytosolic protein S100A9, was statistically significantly identified (Figure 2, Table 1).

### 3.3. Effect of 4-MU on the Secretion of Free Amino Acids during Neutrophil Adhesion to Fibronectin under Control Conditions and in the Presence of LPS

We previously found that neutrophils release branched (valine, isoleucine, and leucine), aromatic (tyrosine and phenylalanine), and positively charged (hydroxylysine, ornithine, lysine, histidine, and arginine) free amino acids during adhesion to fibronectin under control conditions [24,25]. Our data showed that the secretion of only one free amino acid, hydroxylysine, is highly dependent on cell adhesion. Inhibition of neutrophil adhesion through the use of a non-adhesive substrate dramatically and selectively suppressed the release of hydroxylysine [24]. LH inhibitor minoxidil, the MMP inhibitor doxycycline, the PI3K/Akt pathway inhibitors wortmannin and the Akt1/2 inhibitor and actin depolymerizing microbial alkaloids [25], and imipramine [47] blocked the release of hydroxylysine. In all cases, inhibition of hydroxylysine occurred in a selective manner and was accompanied by blocking of cell adhesion and spreading. The release of other free amino acids was stable and did not depend on neutrophil adhesion.

We studied the amino acid profile of neutrophil secretion during fibronectin adhesion under control conditions or in the presence of 4-MU. Neutrophils were stained with trypan blue after removing the extracellular medium for amino acid analysis. The percentage of stained (dead) cells did not exceed 1–2% for control or 4-MU-treated cells, which indicates that the appearance of free amino acids in the extracellular medium is not the result of cell destruction.

**Figure 3:**
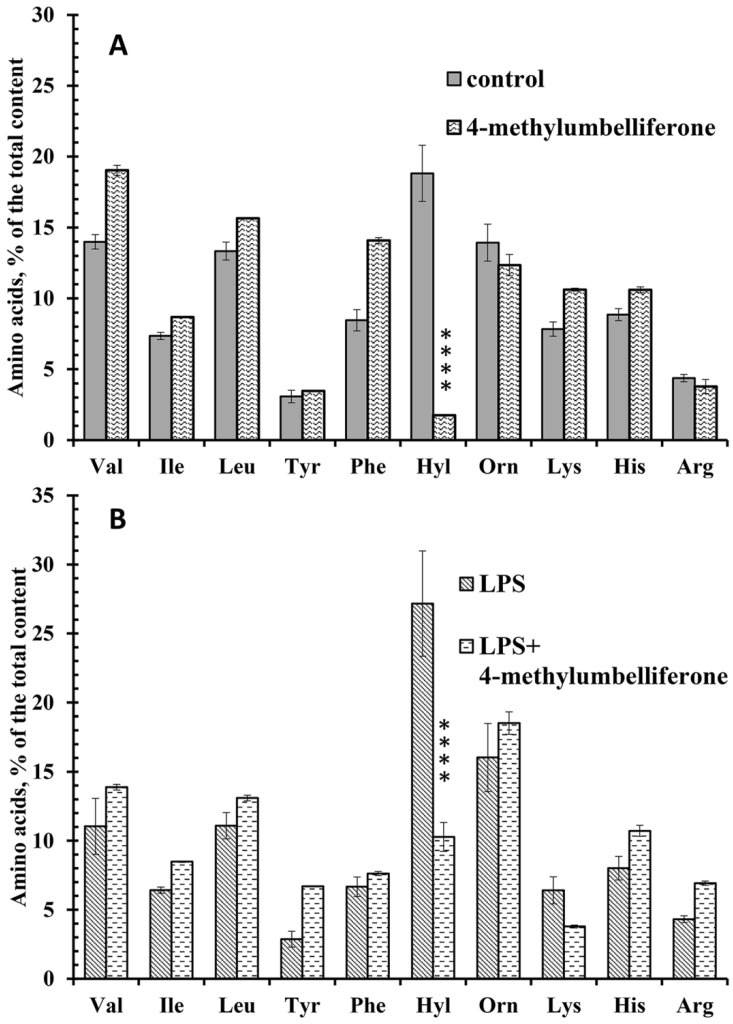
The effect of 4-MU on the free amino acid secretion by neutrophils during adhesion to fibronectin. Neutrophils were attached to fibronectin-coated substrata for 25 min under control conditions or in the presence of 1mM 4-MU (**A**). (**B**) Neutrophils were attached to fibronectin-coated substrata for 25 min in the presence of 10 μg/mL LPS or in the presence of 10 μg/mL LPS and 1mM 4-MU. The amount of amino acid is represented as a percentage of the total content of the detected free amino acids (mean ± SEM). Amino acid profiles were obtained by summing the results of three independent experiments. **** significant differences when compared to the value for the same amino acid under control conditions (**A**) or in the presence of LPS only (**B**) (*p* < 0.0001) as indicated by a two-way ANOVA with a Tukey’s multiple comparisons test.

4-MU selectively and significantly suppressed the release of hydroxylysine, but did not have a statistically significant effect on the release of other amino acids by neutrophils during adhesion (Figure 3A). Our previous data showed that LPS significantly stimulated the release of hydroxylysine by neutrophils upon adhesion to fibronectin, but did not alter the secretion of other free amino acids [47]. In the present work we demonstrate that 4-MU significantly inhibited the release of hydroxylysine by neutrophils that adhere to fibronectin in the presence of LPS, but did not cause statistically significant changes in the content of other amino acids (Figure 3B). Taken together, these data demonstrate that 4-MU selectively inhibits adhesion-induced release of hydroxylysine by control and LPS-treated neutrophils.

### 3.4. Inhibition of NO- and Staurosporine-Induced Cytoneme Formation in Neutrophils by 4-MU

Secretory processes of neutrophils occur by a variety of mechanisms, including the formation, growth, and shedding of cytonemes [41]. Neutrophils can form very long filamentous processes on their surface—cytonemes (cytoplasmic threads). Cytonemes consist of interconnected membrane tubules and vesicles of the same diameter (150–250 nm, depending on conditions), arranged in a row. Cytonemes contain bactericides of primary and secondary secretory granules of neutrophils, that is, they are secretory structures of neutrophils [35,38]. NO produced by neutrophils, endothelial, and other host cells, as well as actin-depolymerizing microbial alkaloids, appear to be natural agents that initiate cytoneme formation in neutrophils [39,40,43]. Both exogenous NO produced by endothelium and other tissues [48], as well as endogenous NO produced by constitutive neuronal type NO synthase presented in neutrophils [49], could initiate the formation of cytonemes. A strong positive correlation has been demonstrated between neuronal type NO synthase and synthesis of stromal HA in breast cancer cells [50]. These data can also support our assumption that synthesis of HA contribute to the NO-induced cytoneme formation. Actin-depolymerizing alkaloids can induce cytoneme formation by stimulating NO production. As shown for platelets and endothelial cells, monomeric actin, formed as a result of depolymerization of filamentous actin, but not filamentous actin, can directly bind to NO synthase, thereby stimulating the production of NO [51,52,53].

**Figure 4:**
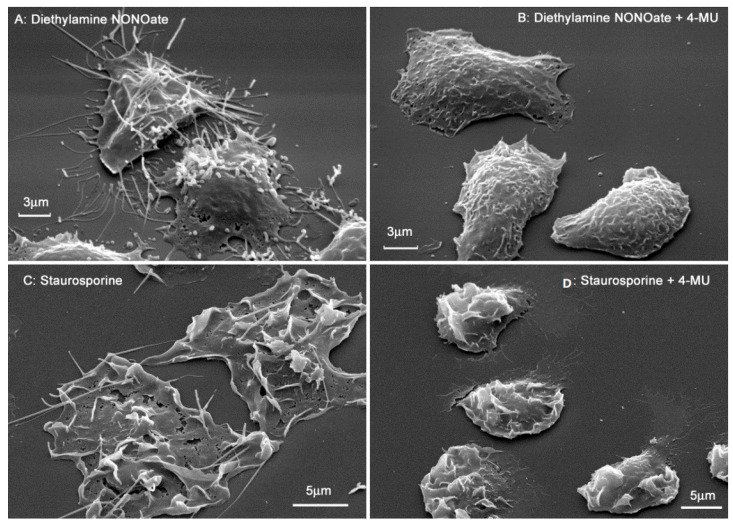
4-MU inhibits the formation of cytonemes in neutrophils in the presence of the NO donor diethylamine NONOate or the microbial alkaloid staurosporine. Scanning electron microscopy of human neutrophils attached to fibronectin-coated substrates for 25 min in the presence of 1 mM diethylamine NONOate (**A**); 1 mM diethylamine NONOate plus 1mM 4-MU (**B**); 200 nM staurosporine (**C**); and 200 nM staurosporine plus 1 mM 4-MU (**D**). Images are typical images observed in three independent experiments.

We studied the effect of 4-MU on cytoneme formation induced by the NO donor diethylamine NONOate or staurosporine. Filiform membrane extensions developed on the surface of neutrophils during adhesion to fibronectin for 25 min (Figure 4A,C). These extensions (cytonemes) were tubular or tubulovesicular in the presence of an NO donor, or tubular in the presence of staurosporine. Cytonemes induced by NO and staurosporine attached neutrophils to the substrate and connected to other neutrophils, thus maintaining cell adhesion and intercellular communication. When neutrophils attached to fibronectin in the presence of 4-MU, cytonemes did not form on the neutrophil surface (Figure 4B,D).

## 4. Discussion

The therapeutic application of 4-MU is associated with its well-established potency to inhibit the proliferation, migration and invasion of multiple cancer cell types [17,18]. Another point of therapeutic application of 4-MU appears to be inflammatory processes [20,21,23]. To clarify whether the anti-inflammatory effect of 4-MU is associated with suppression of neutrophil invasion and neutrophil-induced inflammation, we examined the effect of 4-MU on neutrophil adhesion to fibronectin and associated secretion. Our data showed that the anti-inflammatory effect of 4-MU may be associated with the suppression of secretory processes in neutrophils that accompany and mediate neutrophil adhesion and initiate inflammation. Our data showed that 4-MU inhibited protein secretion by neutrophils during adhesion to fibronectin (Figure 2, Table 1), adhesion-induced release of the free amino acid hydroxylysine (Figure 3), and the formation of cytonemes, extracellular secretory protrusions containing bactericides (Figure 4).

The anti-inflammatory effect of 4-MU does not appear to be associated with inhibition of oxidative neutrophil metabolism. The pro-oxidant effect of 7-hydroxycoumarins was observed in human neutrophils stimulated with PMA or serum opsonized zymosan by chemiluminescence assay with luminol enhancement. The effect was associated with the oxidation of hydroxylated coumarins by neutrophil myeloperoxidase with the formation of highly reactive radical coumarin intermediates [54].

HA associated with the cell surface mediates and modulates the initial stage of cell interaction with the extracellular matrix [55]. HA can influence cell adhesion by modulating the formation of fibronectin and collage fibers, which form the extracellular matrix [56]. The ability of HA to induce the expression and activation of MMPs, enzymes known to modify the extracellular matrix and basement membrane, can also promote cell invasion and adhesion [57,58]. Our previous data revealed that the adhesion-induced release of hydroxylysine was tightly associated with both lysyl hydroxylase (LH) and MMP activity [25]. The mechanism of interaction between LH and MMP in the process of hydroxylysine release is not studied. Some MMP proteolytic activity is observed on the cell surface in the complex with a HA receptor CD44. This interaction provides a mechanism for MMP-mediated tumor invasion [59,60]. Recently, it has been shown that LH3 is also a molecule responsible for recruiting and immobilizing MMP-9 on the cell surface [61]. Drawing an analogy with tumor cell invasion, we hypothesize that interactions between LH and MMP-9 play an important role in neutrophil invasion. 4-MU can affect neutrophil invasion and adhesion through blocking HA synthesis and HA-induced MMP expression [62,63].

How can 4-MU influence various secretory processes in cells through inhibition of HA synthesis? Secretory processes in neutrophils are realized by numerous mechanisms including shedding of membrane vesicles from the plasma membrane. Neutrophils generate functionally and morphologically distinct populations of extracellular microvesicles [64], including integrin-dependent cleavage of extracellular vesicles with antibacterial ability [65]. As shown by electron microscopic examination, their diameter varied mainly in the range of 200–300 nm, which basically coincides with the diameters of NO- or staurosporine-induced cytonemes in our works. We have previously shown that cytonemes contain bactericidal agents of primary and secondary granules of neutrophils [35,38]. The secretion of antibacterial vesicles and the formation of cytonemes appear to be two modifications of the secretion of bactericidal agents by neutrophils. Inhibition of the separation of secretory membrane vesicles from the plasma membrane of neutrophils and from other vesicles may be a key element in cytoneme formation. We hypothesize that NO or actin-depolymerizing alkaloids interfere with membrane fusion/division processes, which are necessary for the separation of secretory vesicles from the plasma membrane and from each other. As a result, the secretory process moves out of the cells in the form of cytonemes—filamentous structures consisting of membrane vesicles and tubules of the same diameter, lined up in a row [37,38].

**Figure 5:**
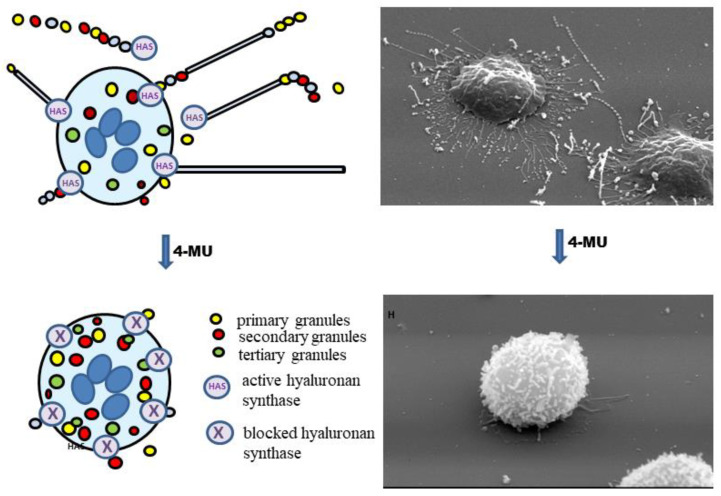
Scheme demonstrates the HA-promoted release of exocytotic secretory carriers from cells and inhibition of this process by 4-MU.

We assume that HA, due to the peculiarities of synthesis, can interconnect vesicular and tubular secretory carriers into cytonemes and provide “extrusion” and detachment of secretory vesicles, tubules, or cytonemes from cells (Figure 5).

The synthesis of HA, linear polymers composed of disaccharides of glucuronic acid and N-acetylglucosamine, occurs in the plasma membrane. Isoforms of HA synthases (HAS1, HAS2, and HAS3) simultaneously lengthen, bind, and extrude the growing HA chain directly into the extracellular space. HA polymer chains are retained on the cell surface by HAS, which, after completion of synthesis, can exfoliate from the cell surface [66,67]. Our assumption that HA can be confirmed by the data that the dense arrays of HA chains, tethered to HAS3 during biosynthesis, can induce and maintain prominent microvilli in adenocarcinoma cells transfected with HAS3 [68]. Elongated microvilli have a length of 3–20 µm and diameter 130 nm, comparable with the length and diameter of cytonemes of neutrophils [41]. Moreover, the tips of long microvilli induced in cells by overexpression of HAS3 were detached into the culture medium as microvesicles [69].

## 5. Conclusions

We demonstrated that inhibitor of HA synthesis 4-MU blocked the secretory processes in neutrophils accompanying neutrophil adhesion to fibronectin. We suggest that hyaluronic acid, due to peculiarities of synthesis, might play an important role in the process of translocation of secretory carriers (membrane vesicles, tubules, or cytonemes) and their content from the cells to the extracellular medium. The anti-inflammatory effect of 4-MU is associated with blocking secretion supporting neutrophil invasion and adhesion to the tissues, as well as secretion of aggressive bactericides responsible for inflammation. The protective effect of 4-MU on non-infection inflammation upon renal ischemia reperfusion injury was demonstrated in mice [22,23]. We suggest that the therapeutic use of 4-MU has good prospects for the treatment of inflammation in metabolic imbalances, in the development of which neutrophil invasion and aggressive secretion play an important role.

## Figures and Tables

**Table 1:** List of proteins secreted by neutrophils when adhered to fibronectin in the presence of 4-MU. Neutrophils were incubated over fibronectin coated substrates for 25 min under control conditions; in the presence of 1 mM 4-MU; and in the presence of 10 μg/ml LPS plus 1 mM 4-MU. Proteins were separated using SDS-PAGE and identified by mass spectrometric analysis. Protein scores greater than 68 are significant (*p* < 0.05). Mass spectrometric data were taken from experiments with 4-MU. Similar proteins identified in control experiments are marked with a (+) sign. The list includes proteins reliably identified in three similar experiments.

	Protein Name			Peptides Matched/ Total	Coverage %	MOWSE Score
	Control	4-MU	LPS + 4-MU			
*Granular proteins*						
PERM_HUMAN	+	MPO	−	18/27	22	118
TRFL_HUMAN	+	LF	−	22/41	36	140
ALBU_HUMAN	+	albumin	−	12/26	19	69
NGAL_HUMAN	+	NGAL	−	7/18	34	73
*Cytosolic proteins*						
ACTB_HUMAN	−	actin	−	12/17	42	153
S10A9_HUMAN	+	S100-A9	+	11/30	73	126
S10A8_HUMAN	+	S100-A8	−	8/32	56	116
